# Extending the Battery Life of the ZigBee Routers and Coordinator by Modifying Their Mode of Operation

**DOI:** 10.3390/s20010030

**Published:** 2019-12-19

**Authors:** Domingo Marrero, Alvaro Suárez, Elsa Macías, Vicente Mena

**Affiliations:** 1 Grupo de Arquitectura y Concurrencia (GAC), Instituto Universitario de Ciencias y Tecnologías, Cibernéticas (IUCTC), Universidad de Las Palmas de Gran Canaria (ULPGC), 35017 Las Palmas de Gran Canaria, Spain; alvaro.suarez@ulpgc.es (A.S.); elsa.macias@ulpgc.es (E.M.); vicenteefigenio.mena@ulpgc.es (V.M.); 2 Departamento de Ingeniería Telemática (DIT), Universidad de Las Palmas de Gran Canaria (ULPGC), 35017 Las Palmas de Gran Canaria, Spain; 3 Departamento de Señales y Comunicaciones (DSC), Universidad de Las Palmas de Gran Canaria (ULPGC), 35017 Las Palmas de Gran Canaria, Spain

**Keywords:** wireless sensor networks, battery life, Internet of Things, sensors, routers, Coordinator, ZigBee

## Abstract

Wireless sensor networks proliferate more and more in all social scopes and sectors. Such networks are implemented in smart homes, smart cities, security systems, medical resources, agriculture, automotive industry, etc. Communication devices and sensors of such networks are powered with batteries: the enlarging of battery life is a hot research topic. We focus on wireless sensor networks based on ZigBee technology. While sleep standard operation mode is defined for end devices, it is not the case for the rest of devices (routers and Coordinator), which usually always remain in active mode. We designed a formal optimization model for maximizing the enlarging of the battery life of routers and Coordinator, allowing us to delimit practical successful conditions. It was successfully tested with a standard ZigBee datasheet comprising technical data for sensors, routers, and coordinators. It was tested in a practical wireless sensor network assembly with XBee S2C devices. We derived, from the previous model, a novel but simple protocol of communication among routers and coordinators. It was tested in different use cases. We showed that when end devices generate traffic at regular intervals, the enlarging of the battery life of routers and Coordinator was possible only under certain use cases.

## 1. Introduction

Wireless Sensor Networks (WSN) have been deployed in many domains such as agriculture, smart cities, smart cars, etc., remotely sensing physical parameters and communicating them wirelessly to an Internet server using Internet of Things (IoT) protocols and services. Different technologies and standards have been developed for the physical and link levels of WSN: nRF24, Bluetooth Low Energy (BLE5.0), IEEE 802.15.4 [1], IEEE 802.15.1 [2], Low Range Wide Area Network (LoRA/LoRaWAN) [3], etc. ZigBee [4] deserves special mention because it is a well-known and commercially established standard. It supports different network topologies, routing, and others network functions and interconnection with IEEE 802.11 [5,6,7,8], commonly known as Wireless Fidelity (WiFi) [9] and the Long-Term Evolution (LTE) technology [10]. 

When a WSN is deployed in zones where the power grid cannot feed it, it must be powered with batteries taking advantage of low consumption WSN devices. ZigBee specifies the firmware of sensors, named end devices (EDs), with different sleep modes. The EDs are programmed to realize a prefixed sampling period, from a few seconds to days or more, and they remain idle until the next sampling period. Conversely, all other devices, like Router Devices (RD) and the Coordinator (C), remain active always. That is, they never are in sleep mode.

In our previous work [11,12,13], we proposed a sleep mode of operation for WiFi Access Points (AP) for regular emitted traffic from WiFi terminals obtaining a considerable amount of energy-saving. Despite WSN and WiFi networks having different characteristics, operations, and functions, in this paper, we apply those previous ideas to include the sleep mode of operation in RDs and C when EDs emit traffic periodically. At the beginning of each period of time, the RDs and C enter active mode and find out the WSN spanning tree using any routing algorithm. The leaf nodes of that tree are the EDs, the intermediary nodes are the RDs, and the root is the C. Each RD manages one branch of the tree and can estimate when it will receive data frames from its children nodes (EDs or others RDs in the tree). After that, the RDs will send data frames to the upper-level RD or to the C (at level 0 of the tree). Once received and after sending the data frames, the RDs or the C can pass to sleep mode until the next beginning of the period of time. The enlarging of their battery life corresponds to the amount of time the RDs and C will be in sleep mode. The main objective of this paper is to show, under previous assumptions, if it is possible to obtain a significate enlarging of the battery of ZigBee RDs and C considering commercial devices. Up to our knowledge, we are the first to consider such an approach, which can be used with any routing algorithm and considers the WSN spanning tree for assuring the forwarding of data messages to the Cloud.

The main contributions of this paper are:

- We introduced the novel sleep mode of operation of ZigBee RDs and C. The importance of this was that we could apply the duty cycle technique and a particular idle time scheduling strategy to enlarge the RDs and C battery life. Theoretical and simulation works on WSN lifetime estimation have often focused on other aspects, but not in the derivation of simple actions in RDs and the C for implementing a simple protocol of communication in real devices, such as the one we designed.
- We designed a novel optimization formal model for estimating the enlarging of RDs and C battery life. It considered the amount of EDs in the WSN spanning tree, the messages length, the cost of wireless communication and routing, and overhead of communication experimented by devices of the WSN. Guided by that model, we derived novel pseudocode of WSN devices that showed how they contemplated the above costs. The importance of the pseudocode was its simplicity to be implemented in a wide range of WSN devices.
- We demonstrated that it is possible to enlarge the RDs and C battery life instantiating our formal model with synthetic data from the ZigBee datasheet of devices, using a simulator and with practical experiments assembled with XBee S2C devices. The importance of these results was that it was not obvious whether the enlarging of battery life will always be possible when using our approach. Comparison with other authors’ methods and our target WSN without our approach showed that the enlarging of the RDs and C battery life can be very significant in certain cases.

The rest of the paper is organized as follows. In Section 2, we review some related work in the scope of enlarging battery life and energy-saving in ZigBee networks. Section 3 presents the main assumptions to formulate our proposal and the context in which it can be applied. In Section 4, we present our optimization formal model for enlarging the battery life of ZigBee communication devices; the actions of RDs and C to accomplish our simple control data interchange among RDs and C. In Section 5, we present how synthetic values from the ZigBee datasheet of devices verified our formal model and how the simulation and experimental results also verified our formal model; a qualitative comparison with other approaches is also given. Finally, in Section 6, we sum up some conclusions and present future work.

## 2. Related Work

Many additional mechanisms have been proposed to enlarge the battery life of WSN devices and accomplish energy-saving, irrespective of whether the devices are already low consumption. In this Section, we briefly present some of those mechanisms and compare them with our proposal. 

One ED in sleep mode, in general, returns to active mode if: (a) it has reached the maximum sleep time; (b) it polls to the Coordinator; or (c) the Coordinator wakes it up when it queues data frames pending to send. We innovatively propose to reduce the consumption of RDs by setting them in sleep mode during the periods they are inactive.

An interesting survey of different strategies for energy-saving in ZigBee WSNs in the context of IoT is shown in [14]. In general, the analyzed proposals are in three main lines: (a) management of channel and timing (physical level) [15,16,17]; (b) modification of the *Medium Access Control* (*MAC*) standard (MAC level) [18,19]; and (c) control of different parameters to reduce consumption [20,21,22,23,24,25]. Here, we include some implementations of ZigBee-analyzed networks.

In [15] is presented a combined mechanism for selecting different slots of time and channel/frequency to distribute the communications between each peer node. In [16] is proposed a method for energy-efficient periodic communication of devices (powered by batteries) over the ZigBee. They use timing channels for different data priorities; thus, more important data are sent more frequently. In [17], the authors proposed a traffic load-based adaptive node scheduling protocol. They scheduled the active and sleep modes of operations of the nodes, defining zones of coverage to adapt the path to the C. These proposals differ from ours: they try to assign different channels or slots of time per communication while we base our solution in the inactivity periods to increase the enlarging of the battery life of ZigBee RDs and C.

In [18] is presented a review and comparative study of IEEE 802.15.4 operating in beacon synchronization and duty-cycling schemes, which affects directly the energy-saving. They analyzed the effects and limitations of their study in cluster topology networks. IEEE 802.15.4e represents an amendment to the standard IEEE 802.15.4 to enhance and add functionalities. Unlike our proposal, we have not considered the use of the super-frame IEEE 802.15.4e and beacon mode to synchronize communication. On the other hand, our proposal is agnostic to the type of WSN topology and wireless technology (which include devices that cannot be in sleep mode). The authors in [19] proposed a method called *Enhancements for Low-Power Instrumentation DSME Applications* (*ELPIDA*) that improved power consumption without introducing extra overhead or long latency. We focus on RDs and the C, and, for that reason, we do not apply MAC operations modes of IEEE 802.15.4e-2012 like Deterministic and Synchronous Multi-channel Extension (DSME), Time Slotted Channel Hopping (TSCH), and Low Latency Deterministic Net Network (LLDN).

In [20], the authors presented two duty-cycling mechanisms for minimizing consumption in the EDs. They used an external microcontroller for a delay-tolerant network. An implementation oriented to capture data of salinity and temperature of the sea was presented in [21]. The authors designed a simple system with Arduino and special sensors. They managed the power using a watchdog timer to set the microcomputer in sleep mode during a specified interval. A proposal based on an XBee ZigBee device is described in [22]. The authors analyzed the torque in a bicycle with a ZigBee network using the standard sleep mode. That proposal did not modify the standard. Time Division Multiple Access is used in [23] to optimize the number of active nodes minimizing message retransmissions. Like the last works, our approach uses a timer to activate the EDs for transmitting data messages to the RD at which they are associated. The authors in [24] presented a set of novel low-power wireless sensor nodes designed for monitoring wooden masterpieces and historical buildings in order to perform an early detection of pests. They proposed a new architecture with a new routing protocol. They demonstrated the feasibility of cluster-based dynamic-tree hierarchical WSN architecture. All the previous proposals differed from ours in two important aspects: they focused on the EDs and they used a microcontroller. In the last one, the authors proposed to manage energy-saving in the WSN routing protocol by applying sleep mode only in the nodes that were not being used. In [25], it is shown that, theoretically, it was possible to optimize the transmission delay and energy consumption of WSN nodes with the designed packet aggregation routing algorithm. They supposed a particular WSN topology similar to a tree in which redundant links among fathers and sons exist. 

In [26], the authors propose a Pipeline Slot based Fast Rerouting (PSFR) scheme to reduce the delay in duty cycle for circular WSNs with sink nodes as the center. They schedule the communications combining slots at the next hop node which is active at the next slot of the active slot of the previous node. With this scheme it is greatly reduced sleep delay. Theoretically, they bounded the duty cycle for nodes when delay was not optimal. We focused on a particular kind of traffic. We were not interested in a general formal model for dimension duty cycle and focused on a technique applied to practical ZigBee RDs and C. Our proposal is agnostic to the routing protocol and only takes advantage of the information it provides for RDs and C to exchange additional control information in the WSN spanning tree. The authors in [27] proposed the simultaneous use of two fuzzy logic controllers to dynamically adjust the sleeping time and the transmission power of the nodes in order to optimize consumption. We want to highlight [28]: a fuzzy logic-based method that considers the throughput, workload, and battery’s level for managing the amount of time the devices of a WSN could be in sleep mode in the domain of the smart home. Three different heuristic optimizations for the duty cycle of nodes were presented in [29] in order to find optimal sensing of different points of interest, overlapping sensing areas among sensors and setting them in sleep mode to improve WSN lifetime. These last proposals were not directed to the RDs and presented a strategy to determinate the EDs and when they can be set in sleep mode. Those proposals differ from ours in that the C assigned the slot and negotiated with EDs their operation mode. We, in contrast, consider EDs to emit data frames periodically. That is, we consider a particular pattern of traffic and then calculating the conditions under the enlarging of battery life is possible. In a similar way, we do not modify the active mode of operation of RDs and C, but introduce the sleep mode of operation for them (as those proposals did not).

Finally, Dynamic Power Management [30] is not intended for RDs and C (it focuses only on EDs); for example, [31] proposed an analyzer based on the Semi–Markov model for Dynamic Power Management in the event-driven sensor node. As we focus on RDs and C, we do not contemplate Dynamic Power Management.

## 3. Assumptions and Application Domain of Our Proposal

**Figure 1:**
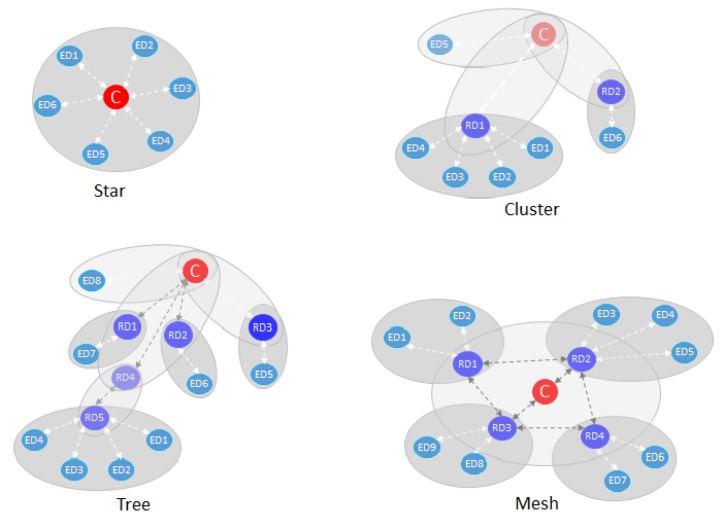
ZigBee topologies.

We apply our proposal to a ZigBee WSN in the context of IoT. At the lowest level, sensors measure physical parameters of interest and, at a high level, those data are stored in the Cloud using IoT protocols and services. Typically, the topology of the WSN is chosen to overcome coverage, traffic rate, capacity, etc. We consider any kind of ZigBee Alliance standardized topologies as indicated in Figure 1. The EDs directly deliver sensed data to RDs or to the C at which they are associated. Our proposal is agnostic to the format and kind of data frame (it can be a ZigBee frame or a packet of any IoT protocol).

Our proposal:

- Does not modify the MAC defined by ZigBee; it does not apply a Time Slot Channel Hopping (TSCH) either, specified in IEEE 802.15.4e;
- Assumes the EDs deliver traffic to RDs or C periodically, according to the timing-driven standard operation mode, when a timer will be reached. That timer is used to wake up the EDs for sending the data frame. While the timer is not reached, the EDs remain in sleep mode. We did not focus on streaming or intensive real-time traffic. If traffic is intensive, critical and streaming is used. It will be sure that our proposal cannot be appropriated;
- Considers it is possible to set in sleep mode the RDs and the C once they have received and forwarded the messages of the EDs and other RDs. We only consider traffic from the WSN to the Cloud;
- Needs the WSN Administrator to program the above timer;
- Supports two kinds of timer specification and programming:
  ○ A unique (shared) timer for all the EDs, RDs, and the C. That means all the end devices simultaneously start to send data at the same time. This generates collisions in the access to the shared wireless channel;
  ○ One internal timer for each ED, RD, and the C. The offset of different timers would be also specified by the Administrator in order to avoid collisions in the shared wireless channel. That is similar to use configurations as Super-frame, GTS [1,2] or Time Division Multiplexing (TDM) [32], which have a minority use or are inexistent in many commercial products. In both cases, the frames sent by EDs are received by the RDs or C sequentially; It is very difficult to correctly schedule the high number of EDs using TDM. For that reason, we study the simplest scheme that consists of using a unique timer for all EDs, RDs, and the C.

Our main assumption is that if the timer is much larger than the time needed to receive and forward messages, then RDs, and the C could enter and remain in sleep mode so that their battery life could be enlarged significantly. That is, once the collisions and other problems of the chaotic wireless channel are solved, and the RDs or the C have received and sent their data frames, they will have time to remain in the sleep mode. 

The coordination of EDs, RDs and the C is crucial in our proposal. That is, at the beginning of each sampling interval (controlled by the above unique timer), the EDs will wake up, they will sense data, and they will send them to the RDs or to the C with which they are associated. For this to happen, the RDs and the C must wake up and receive the data sensed by the EDs associated with them. We assume that the EDs should try this process until they receive an Acknowledge (ACK) indicating the data has been received. Therefore, this process may take some overhead time; but, in any case, coordination is achieved.

The maintenance of the signaling of the channels to be used in the WSN is also an important point in our proposal. In ZigBee, every time a C boots and defines a WSN, it chooses a wireless channel for that WSN. We assume that the C always keeps the same channel to define the WSN at the beginning of the successive sampling intervals. In that way, if all EDs and RDs remember this channel, then the network boot time at the start of a sampling interval can be considerably minimized. As far as we know, this is not contemplated in ZigBee; but, it would be very simple to modify the behavior of the RDs and the C so that they could implement it. On the other hand, WSN security must be strengthened to mitigate the effects of attacks at the wireless channel level. That is beyond the scope of this work. 

Our proposal assumes any kind of routing algorithm in the WSN; but, it is important to choose a routing protocol that generates the WSN spanning tree with low overhead. The Ad-Hoc on-Demand Distance Vector (AODV) protocol is usually used in ZigBee. Its on-demand operation mode does not generate much extra traffic to generate the WSN spanning tree and maintains the links between RDs and the C. If the positions of the WSN devices are static, then the time needed to obtain the WSN spanning tree will be very small, especially if the number of RDs are not high. The Low-Energy Adaptive Clustering Hierarchy (LEACH) routing algorithm could be used to optimize the WSN lifetime. In [33] is presented a formal model for WSN lifetime extension for improving LEACH that was simulated considering a theoretical WSN. In [34] is presented another formal model and simulation for WSN lifetime using a variation of LEACH. In [35], the simulation results for balancing the energy consumption of WSN when all the nodes are required to work at the same time was presented. Those papers were very adequate to observe the problems to design an energy-saving efficient routing algorithm. We did not focus on the specification of a formal model for WSN lifetime using an efficient routing algorithm. Our approach could use any of those proposals but, up to our knowledge, they are not used in practical ZigBee WSN.

**Figure 2:**
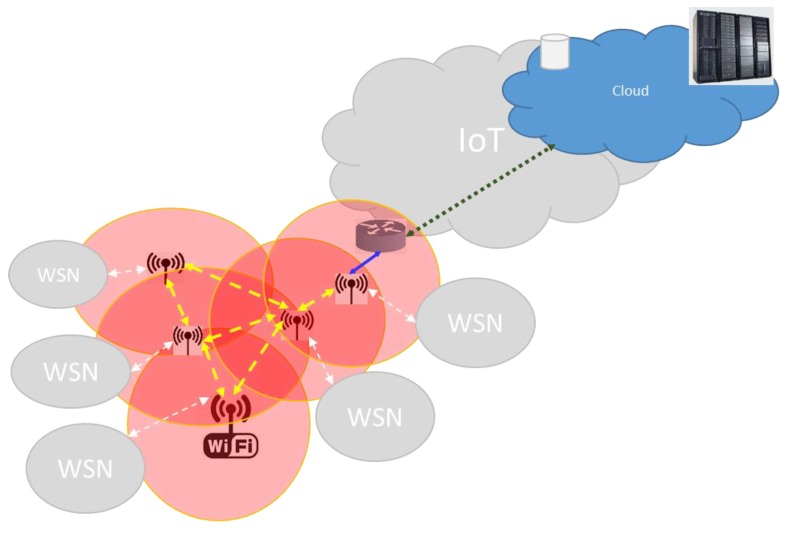
Interconnection of wireless sensor network (WSN) to wireless networks based on different kinds of wireless technologies.

The WSNs usually are interconnected (Figure 2) to a backbone based on other technologies to overcome their limitations, such as wide coverage, high bit rate, capacity, etc. For example, sensing sound in a wide zone of a Smart City synchronously and periodically. For each WSN, a WiFi AP or an Ethernet router can be used to deliver the sensed data to the WiFi or Ethernet backbone. A gateway in each WSN connects with the WiFi AP or Ethernet router: the C of each WSN is in charge to communicate with the WiFi AP or Ethernet router (it can be assisted by a Micro Controller Unit commonly known as Border Gateway). This communication is achieved via a high-speed cable connection, meaning that the reception of WSN messages and forwarding to the backbone can be overlapped in the C. We instead suppose that the C forwards sequentially messages from the WSN to the backbone to keep simple the C. Moreover, one or more WiFi APs (or Ethernet routers) can connect directly to the Internet using Mobile nG technology (2G, 3G, 4G, or 5G). 

We assume the routing in the backbone and Internet does not affect the delay of packets delivered in the WSN. That delay can be mitigated using appropriated buffers in the backbone and Internet and no pressuring backward in the WSN. Therefore, we focus on the data traffic in the WSN. The direction of data traffic is unidirectional from the EDs to the Cloud.

## 4. The Proposal for Battery Life Enlarging for RDs and the C

We first present the formal model of our proposal, then we will bind the formal model with realistic assumptions and, finally, we will present the protocol and additional behavior of RDs and the C in a WSN for supporting our proposal.

Let us suppose that in the WSN there are *n* RDs and *e* EDs. After the WSN spanning tree is formed by the routing algorithm (at the beginning of the sampling period), the messages sent by the EDs must traverse the WSN spanning tree to reach the C. 

Taking into account the assumptions of the previous Section, let us define the following parameters:

- *t_s_*: the unique timer for all the devices in the WSN. That timer is the sampling period (time between one measure, the sending/receiving of the data frame and the following measure). The communication devices do not use it for global synchronization. The different devices evolve independently and are coordinated by message passing, as in ordinary WSNs. It must be prefixed in the configuration of devices by the Administrator of the WSN to determinate the timing between measures. Its value depends on the kind of application and domain in IoT;
- *T*: a period of time in which we study the enlargement of the battery life of the RDs and the C. The *T* is related to *t_s_* by the expression: $T=k*t_{s}$. That is, the *T* is a positive integer multiple (*k >* 0) of *t_s_*;
- $M\;\left(m_{0},m_{1},\;\ldots ,\;m_{n}\right)$: a vector whose elements contain the number of EDs directly associated to *RD*_0_, *RD*_1_, …, *RD_n_*_−1_ and the *C*, respectively, in the WSN spanning tree (computed each *t_s_* units of time by the routing protocol); where *n* is the amount of RDs and $e=\sum_{i=0}^{n}m_{i}$ is the amount of EDs in the WSN;
- $L\;\left(l_{1},l_{2},\;\ldots ,\;l_{e}\right)$: a vector whose elements contain the length of the messages sent by the EDs (as previously assumed, we consider each ED sends only one data message in each *t_s_*). Typically, the length of the messages is fixed in ZigBee or the particular IoT protocol. Let *r* be the bit rate of the common wireless channel, then the transmission time of the messages sent by the EDs can be calculated as: $Tx\;\left(tx_{1},tx_{2},\;\ldots ,\;tx_{e}\right)=\frac{L}{r}=\left(l_{1}/r,l_{2}/r,\;\ldots ,\;l_{e}/r\right)$.

In a *t_s_* interval of time, each ED spends an amount of time sending their messages to the RDs at which they are associated. The RDs must receive the messages from their associated EDs and forward them to their RDs parents in the WSN spanning tree. Finally, the C receives the *e* messages from their RDs children and associated EDs and then it will send them to the Cloud (using the connection to the WiFi AP or Ethernet router). Finalizing the above communications (in each device), they can be set to sleep mode. Theoretically, the time *EDi* spends sending its message to the RD is $tx_{i}$ and the time spent by the C in receiving messages is $\sum_{i=1}^{e}tx_{i}$. The communication time of RDs depends on their number of RDs children and associated EDs in the WSN spanning tree.

**Figure 3:**
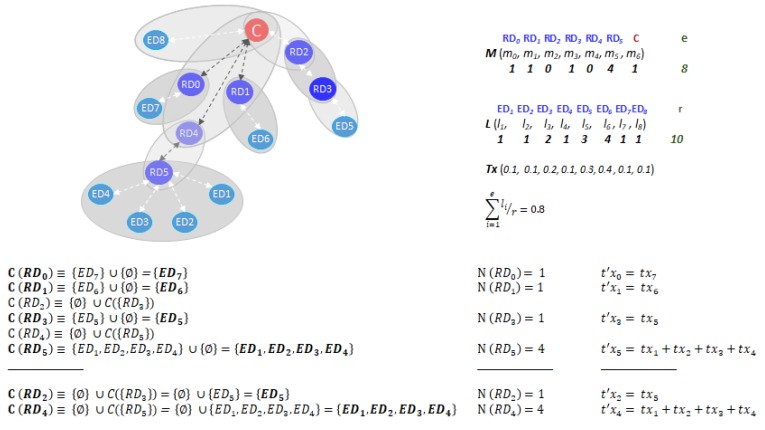
Calculation of *C*(*RD_i_*), *N*(*RD_i_*), and *t’x_i._*

Let C be the set of EDs children of the *RD_i_* (*i* = 0, 1, ..., *n* − 1) given by the union of the set of EDs associated with it and the set of its *RD_k_* (k≠i) children. That is, $C\;\left(RD_{i}\right)\equiv \;\left\{ED_{r},\;r\in \left\{1,\;2,\;..,\;e\right\}\right\}\;\cup$ $\left\{C(RD_{k}),\;k\neq i,\;i=0..n-1\right\}$. This set is calculated iteratively. Then, the number of messages, *RD_i_* must receive is: $N\;\left(RD_{i}\right)=rank\;\left(C\left(RD_{i}\right)\right)$, where *rank* returns the number of elements of $C\left(RD_{i}\right)$. Thus, the theoretical communication time of *RD_i_* for communicating the messages of its $C\;\left(RD_{i}\right)$ set to its RD parent (or the C) is given by:

(1) $$t^{\prime }x_{i}=\overset{N\;\left(RD_{i}\right)}{\underset{j=1,\;j\in \left\{1,\;2,\;\ldots ,\;e\right\}}{\sum }}tx_{j}$$

where *j* is defined in the set of indexes of children contains in the $C\left(RD_{i}\right)$ set. A graphic example of calculation of the above sets and communication time of RDs are shown in Figure 3.

The RDs must receive the messages from their EDs children and forward them to their RD (or the C) parent in the WSN spanning tree. Thus, its communication time is twice $t^{\prime }x_{i}$. Although ACK messages can be deactivated in ZigBee, our proposal contemplates the worst case for communication time forcing all the messages to be acknowledged. Sending an ACK from an *RD_i_* to its children does not influence $t^{\prime }x_{i}$, but receiving an ACK from its parent does influence $t^{\prime }x_{i}$. Therefore, $t^{\prime }x_{i}$ should include the waiting time for each of the ACK messages that an *RD_i_* receives from its parent in the WSN spanning tree. Typically, the length of ACK messages is much smaller than the data messages’ length. Let 0≤w<1 be the weight that relates the length of a data message to the length of an ACK message. With all this, a more realistic value for $t^{\prime }x_{i}$ is:

(2) $$t^{\prime }x_{i}=2\underset{j}{\sum }tx_{j}+\;\underset{j}{\sum }w\;tx_{j}=\left(2+w\right)\underset{j}{\sum }tx_{j}$$

In practice, the communication of messages experiments overhead due to:

- Contention in the shared wireless chaotic channel, collisions in the channel, interferences among different sensors, RDs, the C, and other polluting wireless devices close to the WSN. These effects produce delays in the sending and reception of messages in WSN devices. In [36], an interesting review of problems with realistic communications due to MAC and interferences affecting the delay of messages communication in a balanced tree-topology WSN was explained. The detailed formal specification of realistic communication conditions in the wireless channel was out of the scope of this paper. In contrast, we were interested in a formal model that guided us to derive the behavior (pseudocode) of WSN devices. In that code, the messages’ communication time was taken into account;
- Buffering time of messages, messages lost, message retransmission, etc.;
- Calculation of the WSN spanning tree at the beginning of *t_s_*.

Let $O\;\left(o_{0},o_{1},\;\ldots ,\;o_{n},\;o_{n+1},\;\ldots ,\;o_{e+n}\right)$ be the vector of overheads of communication in which the elements represent the communication overhead experimented by RDs ($o_{0},o_{1},\;\ldots ,\;o_{n-1}$), the C ($o_{n}$), and Eds ($o_{n+1},\;\ldots ,\;o_{e+n}$). Then, the amount of time an *RD_i_* (or the C) must remain active (on) in a *t_s_* is calculated from Equation (2), as $ton_{i}\;\left(i=0,\;1,\ldots ,n\right)$:

(3) $$ton_{i}=o_{i}+\left(2+w\right)\underset{j}{\sum }tx_{j}$$

Thus, the amount of time an *RD_i_* (and the C) in a *t_s_* can be set to sleep is given from Equation (3) as $s_{i}\;\left(i=0,\;1,\ldots n\right)$:

(4) $$s_{i}=t_{s}-ton_{i}$$

**Figure 4:**
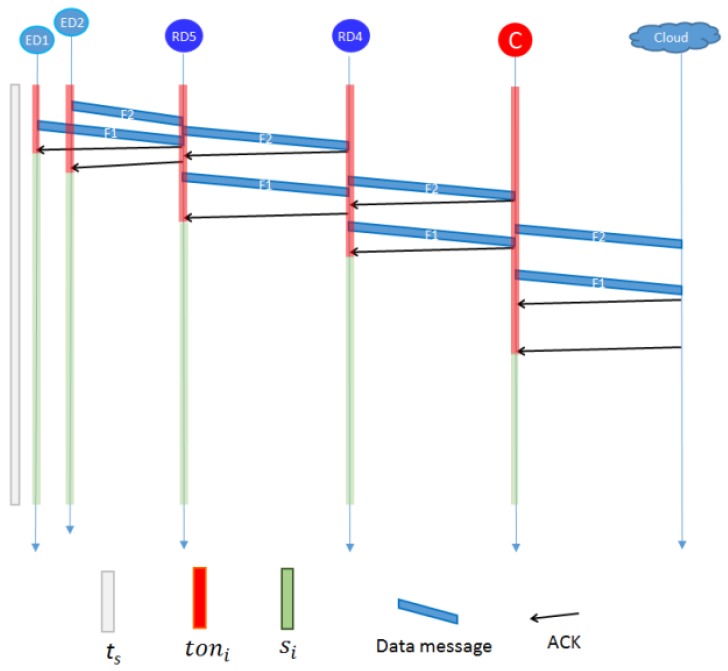
Graphic idea of *Ton* and *S* in the end devices (EDs), router devices (RDs), and Coordinator (C).

Figure 4 shows an example of graphical values of above parameters for the *ED*1, *ED*2, *RD*4, *RD*5, the *C*, and the *Cloud* of Figure 3. Once EDs receive the ACK, they can be set on sleep mode. Let us note that the ACK arrives to the *ED*2 later than the arrival of ACK to *ED*1. The *ED*2 starts the communication before than *ED*1. This is due to the overheads of wireless communications. The *RD*5 cannot enter sleep mode until it receives the ACK from *RD*4. Since *RD*4 has no associated ED, it simply forwards the messages to C, waits for ACKs, and enters sleep mode. If it had associated EDs, it must wait also for their messages to be sent before entering sleep mode.

The calculation of the $tx_{j}$ and the value of w, as well as the estimation of $o_{i}$ (its variability due to the chaotic nature of wireless channels making it very difficult or impossible), is important because $s_{i}$ depends on it. From Equation (4), it is directly observed that $t_{s}>ton_{i}$ because otherwise the *RDi* (or the C) could not enter sleep mode. 

Estimation of the maximum value of $tx_{j}$ and w can be done from the datasheet of manufacturers. For determining a practical value of them, we did some practical tests with real products (XBee S2C [37]) measuring real traffic into a WSN prototype with the Texas Instruments sniffer ZB CC2530 [30] analyzer. We checked that these products included several analog and digital inputs to sample different physical parameters. So, we know that the size of data frames depends on the number of active analog and digital inputs. The number of bits of the different samples was different (10 *b* for an analog to digital conversion, 8 *b* for digital data). We considered an intermediate size where it only used four digital inputs and one analog input. In that case, the longest size, L=Max (L), of those frames was of 71 B. That makes *L* = 71 [*B*] * 8 [*b*] = 568 *b*. The ZigBee devices theoretically operated at r=250 kbps, thus $tx_{j}=568\;b=250,000\;\mathrm{bps}=0.0022\;s=2.2\;\mathrm{ms}$.

There is a direct relation between $s_{i}$ and the enlargement of the battery life of *RD_i_*: the bigger $s_{i}$ the bigger the enlargement of the battery life of *RD_i_*. If the *RD_i_* is fed by a voltage $V_{i}$ and consume $I_{i}$ amperes, its *Power* consumption (*P_i_*) in the period of study *T* will be (taking into account Equation (4)):

(5) $$P_{i}=\;\int_{0}^{T=k*t_{s}}V_{i}\left(t\right)\;I_{i}\left(t\right)\;dt=\;\int_{0}^{k*s_{i}}V_{i}^{S}\left(t\right)\;I_{i}^{S}\left(t\right)\;dt+\int_{0}^{k*ton_{i}}V_{i}^{O}\left(t\right)\;I_{i}^{O}\left(t\right)\;dt\;\;$$

where $V_{i}^{S}$ is the feed voltage when the *RD_i_* will be in sleep mode, $I_{i}^{S}$ is the amperes consumption when the *RD_i_* will be in sleep mode, $V_{i}^{O}$ is the feed voltage when the *RD_i_* will be on (active), and $I_{i}^{O}$ is the amperes consumption when the *RD_i_* will be on (active). Typically, the values provided by the manufacturer are fixed and are such that $V_{i}^{S}<V_{i}^{O}$ and $I_{i}^{S}<I_{i}^{O}$. Thus, it is crucial to maximize the amount of time the *RD_i_* will be in sleep mode ($s_{i}$) to enlarge its battery life. That is, from Equation (4), $MAX\;(t_{s}-ton_{i})$. From, Equation (3), $MAX\;(t_{s}-(o_{i}+\left(2+w\right)\underset{j}{\sum }tx_{j}))$ requires minimizing which is equivalent to make $MIN\;(o_{i}+\left(2+w\right)\underset{j}{\sum }tx_{j})$ and, at the same time, $\;MAX\;(t_{s})$ must be accomplished. Unfortunately, $o_{i}$ is very difficult to estimate and w and $tx_{j}$ are parameters that cannot be easily modified because the firsts depend on the standard ZigBee and the second depends on the physical parameters to be measured and the ZigBee standard or other IoT protocols. Moreover, the optimization of all the batteries’ life (i=0, 1,…,n) is a very hard problem to be solved analytically. For that reason, we first study the relation between *t_s_* and the above parameters, and then we present a protocol for allowing the RDs and the C to enter sleep mode (enlarging in that way their battery life). 

**Figure 5:**
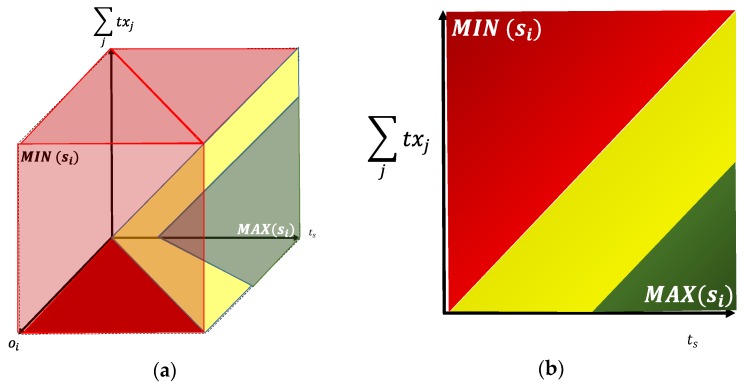
Color graphic for bounding the values of ***S***: (**a**) Three dimensions. (**b**) Two dimensions.

Figure 5a shows a color graphic with the maximum and minimum values of $s_{i}$, taking into account the components of Equation (3). The red color marks the inappropriate values of $s_{i}$. The green color marks the range of appropriate values. The yellow color marks the appropriate but intermediary values. Considering $\left(o_{i}\ll \left(2+w\right)\underset{j}{\sum }tx_{j}\right)$, in Figure 5b the above regions for two dimensions are shown. In practice, it is important to maintain *t_s_* as much greater than the time needed to communicate messages of EDs in each *RD_i_* (and the C), independently. In that case, the enlarging of RD (and the C) battery life is possible. For doing that, it is necessary to calculate $C(RD_{i}),\;i=0..n$. To do this, M must be distributed among the RDs and the C at the beginning of the *t_s_*. To keep the distribution simple, it is important to obtain a simple and efficient behavior of the RDs and the C to include the sleep mode. 

In Table 1 we show the pseudocode of the RDs and the C for including the sleep mode in their operation (by completeness we present the pseudocode of the EDs). In bold is set the extra code for modifying the behavior of the devices. The function *ReadConfigurationVariables* (*T*, *t_s_*) reads the *t_s_* value provided by the WSN Administrator. The *T* contains the period of time to study the battery life. All the communication devices wait until the Administrator send *T* and *t_s_* via the broadcast, which grants all the communication devices in the WSN to start working simultaneously. That maximizes the performance of our proposal because all communication devices would define *t_s_* (deadline) almost at the same time. As an example, this works in small WSN for agriculture installed in a small-medium area. The function *ReadInternalClock* () reads the internal clock of the WSN device in order to compute the elapsed time. The first instance marks the beginning of the active state of the device and the second one at the end of the active state. Then, the function *Sleep* sets the device in sleep mode for a period of time contained in its argument (the argument is provided by the Equation (4)). It is shown that the behavior of the EDs remain unaltered (only instrumental code is inserted to set them to sleep). The novel behavior of RDs (and the C) include two functions: *WaitSpanningTreeGenerated* () that is in charge of waiting for the routing algorithm to generate the spanning tree and to communicate to the devices; and the *ComputeC*(*RD_i_*) that is in charge of distributing M among the RDs (and the C). Moreover, it computes the list of children EDs and RDs iteratively as indicated in Figure 3. Both functions spend some time. That amount of time must be included in the $o_{i}$ in order to verify the experimental results in the next Section. If an RD (or the C) detects that *t_s_* − (*t*_2_ − *t*_1_) is very small in two or more consecutive iterations of the repeat loop, it could mean that some of its children have a *t_s_* value that expires long after the RD (or the C) *t_s_*. Then, the RD (or the C) checks if some of the RD children takes too long to send its data and eventually the RD will send the next expiration of *t_s_* to those children (using piggybacking in the ACK message). That is the reason the EDs will wait for an ACK. Let us remark that WSN devices support EDs emitting non-critical traffic randomly at different times inside the sampling period, because all of them will be synchronized by message passing. If that happens, the probability that *t_s_* − (*t*_2_ − *t*_1_) will be short is high (our approach will not be efficient).

## 5. Experimental Results Verification

In this Section, we first present a synthetic evaluation of Equation (4) for delimiting *s_i_*. Enlarging of the battery life is directly related to the consumption of current and voltage in the RD: the more time the RD spends in sleep mode, the more enlarging of battery life will be obtained. We determine different use cases for obtaining the better values of *s_i_.* Secondly, we take the better values and show the amount of battery life enlarging using manufacturer specification of commercial devices as XBee S2C [37]. Finally, we test those values with real measurements on those devices.

### 5.1. Synthetic Evaluation of s_i_

We implemented Equation (4) in C language [38], under the following realistic restrictions (Table 2), in order to obtain the average rounded value of *s_i_* (60 sampling periods):

- We considered 1 *RD*.
- We considered five use cases to classify the values of *s_i_*: *Ideal* (all frames perfectly aligned), *Acceptable* (all the frames are not aligned just as they do in WSNs: acceptable communication gaps are generated synthetically), *Acceptable with few amounts of EDs* (reduced *m_i_* because for higher *m_i_* frames could occupy entire sampling period), *Acceptable with restrictions* (short frames), and *Unacceptable* (bad conditions to obtain *s_i_*).
- We fixed *o_i_* = 0 (negligible) for the *Ideal* use case. For *Acceptable* and *Acceptable with restrictions*, we chose *o_i_* =0 or *o_i_* = 1. We chose *o_i_* = 0, 1 or 2 for *Acceptable with few amounts of EDs*, and *o_i_* = 0, 1, 2, or 3 for *Unacceptable* use case. For all use cases (except *Ideal* one), we generated random communication gaps between frames (modeled as integers between 0 and 3).
- For each use case, we considered *m_i_* took the values: 1, 2, 5, 10, 20, and 30.
- We considered discrete values of $t^{\prime }x_{i\;}$ = 1 (minimal frame size), 2, 3, and 5.
- In order to represent the average round of *s_i_* in term of percentage of *t_s_*, we considered in all cases *t_s_* = 100.
- The values of *o_i_*, *t_s_* and $t^{\prime }x_{i}$ were expressed in *time units* [*tu*] in order to abstract real measurement of time in ordinary units like *s, ms, m, h*, etc. In that way, the obtained values can be scaled to any of those units.

When *s_i_* = 0 (0% of time), it is not possible to enlarge battery life. This occurs in different use cases: (a) <*Acceptable* (*few EDs*), *o_i_* = Random in [0–2], *m_i_* = 30, $t^{\prime }x_{i}$ = 1>; (b) <*Acceptable* (*restrictions*), *o_i_* = Random in [0–1], *m_i_* = 10, $t^{\prime }x_{i\;}$ = 5>; (c) <*Un*a*cceptable*, *o_i_* = Random in [0–3], *m_i_* = 20, $t^{\prime }x_{i}$ = 1>; and (d) <*Un*a*cceptable*, *o_i_* = Random in [0–3], *m_i_* = 30, $t^{\prime }x_{i}$ = 1>. That is, when: (1) *m_i_* = 30 (except for the *Ideal* use case), *s_i_* = 0, (2) *m_i_* = 20, $t^{\prime }x_{i}$ =1, *s_i_* = 0, (3) *m_i_* = 20, $t^{\prime }x_{i}$ = 5 (frame length is large), and *s_i_* = 0. These three use cases are not common in real implementation of WSN because they do not guarantee the sending of at least one data frame for each ED in each sampling period. Therefore, when *o_i_* is low (negligible values) and the frames have a minimal size, *s_i_* will be high (battery life enlarging is possible, but we must guarantee that $ts>\;ton_{i}+\;s_{i}$ to enlarge battery life).

**Figure 6:**
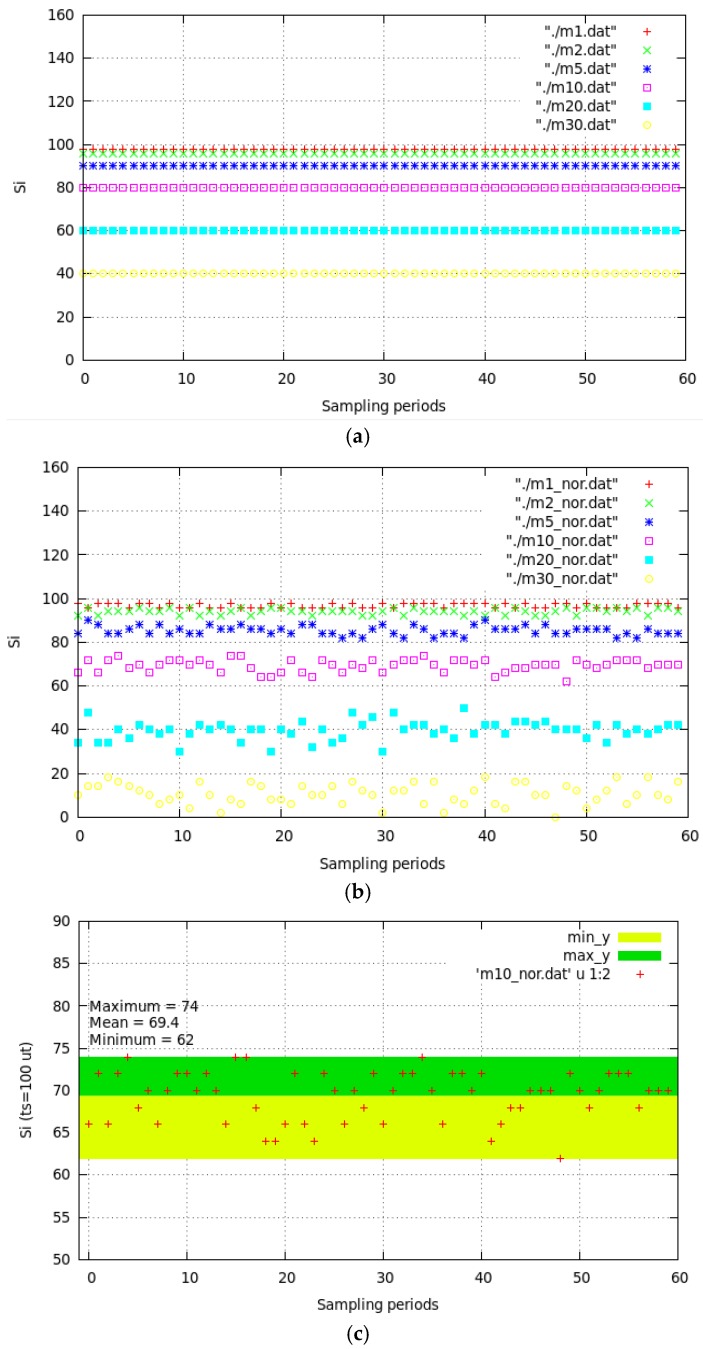
Values of *s_i_* in 60 sampling periods for different *m_i_* values: (**a**) *Ideal* use case; (**b**) *Acceptable* use case; (**c**) mean value for *m_i_* = 10 for *Acceptable* use case.

Figure 6a shows the *Ideal* use case results of simulating for *o_i_* = 0 and Figure 6b shows the results of *Acceptable* use case of simulating random ([0,1]) values for *o_i_*. These figures show the *s_i_* for 60 sampling periods coding the *m_i_* values with colors (red for 1, green for 2, blue for 5, pink for 10, light blue for 20, and yellow for 30). As expected in Figure 6a, *s_i_* reaches it maximum value always. Figure 6b shows values of *s_i_* in the range 98% for *m_i_* = 1, which indicates that the maximum battery life enlarging can be reached: around 92% for *m_i_* = 2 and 85% for *m_i_* = 5. Figure 6c shows the case for *m_i_* = 10 with a minimum (62 *tu)*, maximum (74 *tu*), and mean (69.4 *tu*) values of *s_i_*. That is, the RD will be in sleep mode around 70% of time in each sampling period. The total power consumed for one RD will be inversely proportional to *s_i_*. Concretely, Total RD power = 30% power in active mode + 70% power in sleep mode. This also happens for different values of *m_i_*.

### 5.2. Instantiating Previous Appropriated Values of S_i_ into Commercial ZigBee Devices’ Datasheet

In our laboratory, there are some XBee S2C of Digi devices whose main characteristics (extracted from their datasheet) are shown in Table 3 according to the state in which they could be. They consumed the same current (Amp) independently of the mode they work (ED, RD, or Coordinator). We suppose they are fed with a battery capacity of 1100 mAh. 

We now consider the battery life of an XBee S2C to show the theoretical performance of our battery life enlarging proposal. From the above specifications, and assuming a linear discharge, the following interval limits of an ED (let us note that an ED consumes the same as an RD during receiving or transmitting data) that is always transmitting or receiving data:

1100 mAh/45 mA = 24.5 h ~ *1 day* <= Battery life <= 1.6 *days~* ≅ 39.2 h = 1100 mAh/28 mA (6)

That interval shows that the amount of time an XBee S2C device can be transmitting or receiving is very low. In contrast, the battery life of one ED always sleeping is in the order of hundreds of years: 1100 mAh/0.001 mA = 11 × 10^5^ h. These theoretical results show the importance to force the XBee S2C devices to enter sleep mode the most time possible.

Let us now treat with the worst case. The device transmits in boost mode wasting 45 mA (instead of the datasheet indicates that receiving operation waste less current) and could be in sleep mode wasting 0.001 mA, in order to apply our battery life enlarging proposal. Let us segment each sampling period into two parts depending on the state of the XBee S2C device: active mode (transmitting or receiving) and sleep mode. Concretely, we are interested in the amount of time the XBee S2C device spends in each mode of operation. Let $ton_{i}$ [%] be the amount of time the XBee S2C device spends in active mode in one sampling period and let $\bar{s_{i}}$ [%] be the mean value of *s_i_* calculated in Section 5.1 for each sampling period. If the amount of time of a sampling period represents the 100%, then $ton_{i}$ = 100 − $\bar{s_{i}}$. We highlight that calculating battery life with a high level of accuracy is very difficult, especially when there are different levels of power consumptions that are constantly changing until the total discharge. That calculation depends on temperature, cycles of charge/discharge, internal resistance, initial state of battery charge, etc. Typically, the average amperage (current or discharge rate) is usually used to estimate it. For this, we have to relate the sampling period with the unit of time (hour) normalized for manufacturers (mAh). We have considered the values of $ton_{i}$ and $\bar{s_{i}}$ related with respect to sampling periods of one hour to estimate the battery life. So, we can approximate the amperage average using $ton_{i}^{\prime }$ and ${\bar{S}}_{i}^{\prime }$ in the form:

1 h = 3600 s → [100]

$$ton_{i}^{\prime }\to ton_{i}[\%]$$

$${\bar{S}}_{i}^{\prime }\to \bar{s_{i}}[\%]$$

$$Amperage\;average=\;((45\;\mathrm{mA}\;*\;ton_{i}^{\prime })\;+\;(0.001\;\mathrm{mA}\;*{\bar{S}}_{i}^{\prime }))/3600\;s$$

where we have indicated the amperages in each mode.

Table 4 shows the increasing of battery life is directly proportional to $\bar{s_{i}}$ which clearly indicates the benefits of our proposed battery life enlarging proposal. The battery life enlarging is increased if the duration of $\bar{s_{i}}$ is increased (for comparison purposes, the last row titled No Sleep Mode specified the case in which our proposed approach was not used).

We highlight that in all use cases where $\bar{s_{i}}$ ≠ 0, the battery life is enlarged until high values because we used very low values of amperage in sleep mode (1 uA) taken from the datasheet.

### 5.3. Experimental Power Consumption Evaluation of Commercial ZigBee

In order to compare the results of Section 5.2 with the ones obtained in a real assembly, we made real measures of consumed current, in one XBee S2C device connected to other electronic components. 

**Figure 7:**
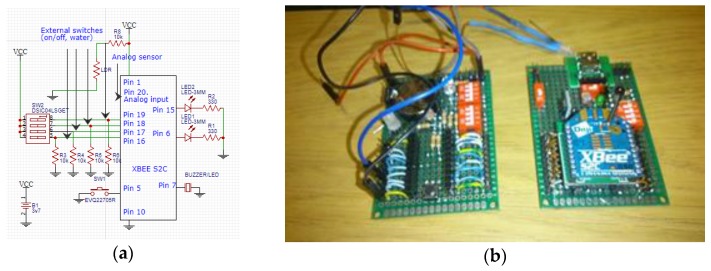
Kits for testing of ED and RD: (**a**) electric sheet; (**b**) real photo of two nodes (router_2 and router_1).

We assembled several XBee S2C devices as a node with additional electronic components (sensors, resistors, light-dependent resistor (LDR), light-emitter diode (LED) to indicate the Received Signal Strength Indication (RSSI) and association (blinking LED). Let us note that these components can increase the current consumption. We named the node with less current consumption router_1 and the one with some higher consumption router_2 (more additional electronic components were attached to router_2 than to router_1). We used a Digital Multi-meter JHS MY-64 [39] to realize the current measurements. To know the state of each device, we added an LED (sleep mode indicator) to monitor them. The LED is on when the EDs are in active mode LED is off when the EDs are in sleep mode. Figure 7a shows the schematic of the electronic kits. Figure 7b shows a photo of two kits with devices XBee S2C. 

**Figure 8:**
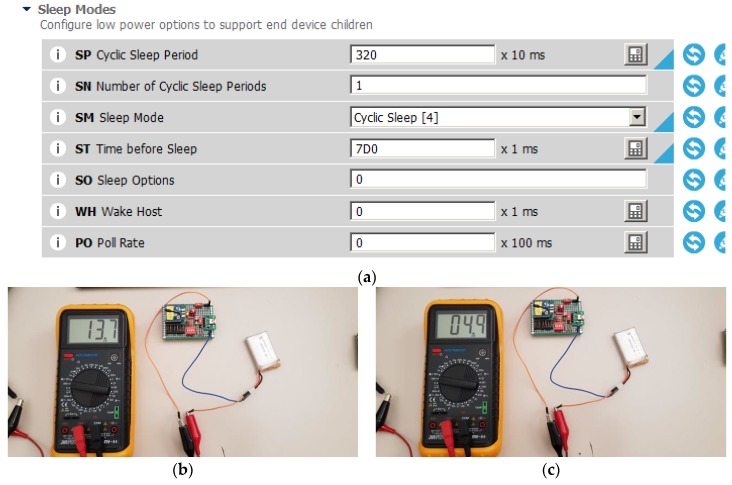
Configuration of XBee module (router_1) and its current consumption: (**a**) timing and sleep mode duration; (**b**) current consumption in active mode; (**c**) current consumption in sleep mode.

We have centered in router_1 to realize the measures and estimations although the results are extensible to router_2 (corresponding consumption will be higher). Figure 8a shows the configuration of XBee module (router_1) with sleep mode activated. Once the module is associated to the C, we measured its current consumption. First, we configured router_1 as a data source with *t_s_* = 10 s, *s_i_* = 8 s, and $ton_{i}$ = 2 s. Its current consumption was 13.9 mA (Figure 8b). Figure 8c shows its current consumption in sleep mode (4.9 mA). Let us note the wide difference of current consumption in sleep mode compared with the 0.001 mA specified in the datasheet of the XBee S2C devices due to the additional components.

According to these results, the worst battery life case for this ED would be: 1100 mAh/13.7 mA ≅ 80.29 h if it always will be in active mode and 224.48 h if it always will be in sleep mode. Then, we can estimate the increase of discharge time depending on $\bar{s_{i}}$ when a node operates as RD. Table 5 shows the results for each case and battery life (for comparison purposes, the last row, titled *No sleep mode*, specified the case in which our proposed approach was not used). As we only are interested in analyzing how the discharge time increases, as we did in Table 4, now we show the estimated battery life considering the time in active mode plus the time in sleep mode. Obviously, the lower amperage in sleep mode will increase the estimated battery life in a corresponding proportion to $\bar{s_{i}}$ (it is expressed in parts per unit). 

**Figure 9:**
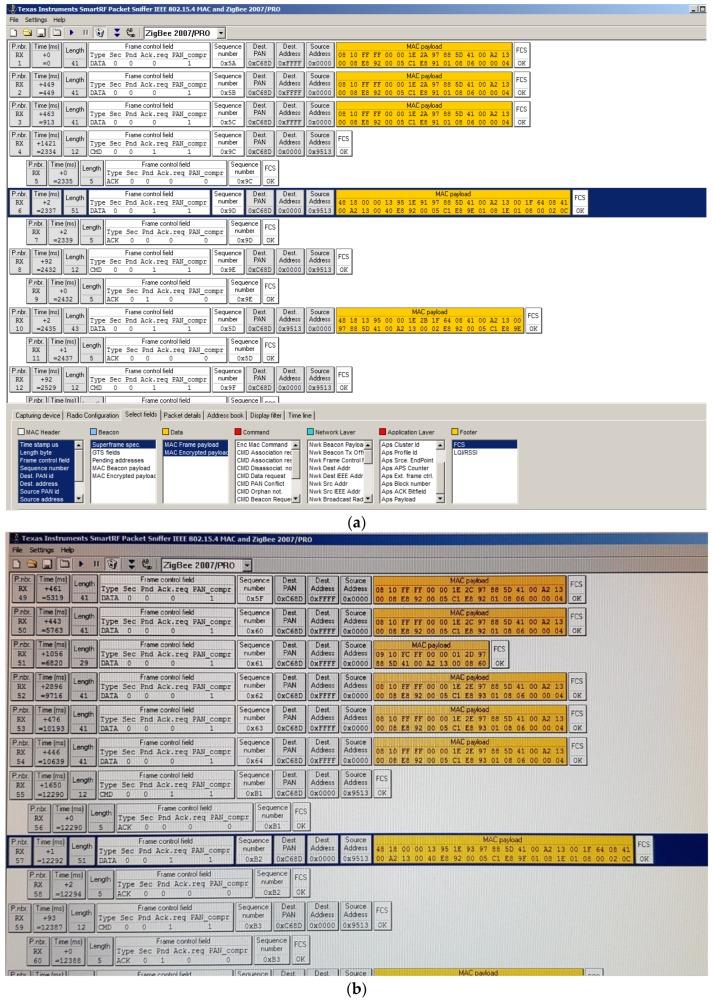
Frames sent for router_1 each 10 s: (**a**) time instant 2.2 s; (**b**) time instant 12.2 s.

Moreover, we sniffed transmitted frames, using the sniffer analyzer presented in [40], in order to test that they were transmitted according to standard specifications. Figure 9a shows captured data in the instant 2.2 s and Figure 9b shows captured data for instant 12.2 s. These values confirm the 10 s of sampling period. The rest of traffic showed corresponds to control frames.

Analyzing Table 5, it is appreciable that if *s_i_* is high, the battery life will be increased proportionally. At the first row (*m_i_* = 1), an increase is shown until six days because the $\bar{s_{i}}$ is the highest. 

### 5.4. Results Discussion

Comparison with other works cannot be done directly because no other work specifies concretely the enlarging of the RDs battery life. Some papers are dedicated to theoretically compute the entire WSN lifetime and others focus on the WSN lifetime using new or modified routing algorithms. We compared different works that did not propose directly the enlarging of EDs battery life. Those methods are [20,22,24,27,30]. We rule out doing a complete simulation of the other methods because we did not have the essential details to be able to do it. We also ruled out its practical implementation because we did not have the necessary material for doing it in our laboratory. For those reasons, we made an approximate qualitative comparison.

All the methods used one C, but the majority of them did not use a battery (it was complicated to do an accurate comparison). Only [22] explicitly used one RD for experimentation (we used one C that also made the function of RD). The rest of the compared works used a flat WSN (all devices were considered as EDs). That also complicated an accurate comparison because we focused on the enlarging of RDs battery life but those works were focused on WSN nodes. The tree-topology was used by [22,24] and us (one-level tree). The rest of the methods used star topology ([24] also used a cluster topology). One-level trees and star topologies can be compared under certain kind of traffic (and conditions) and behavior of the WSN devices. But the problem was that the target kind of traffic was not specified in [27,30]. The number of EDs used by the methods were: one in [20,22] and our method (although we have presented previously results for more EDs); four in [27,30], and 10 in [24]. The technology testbed also differed among methods. In Table 6, we present a qualitative comparison of those methods with ours. The column named Battery Life Specified in Each Paper for Each ED was taken from each compared paper directly. In our method, this was not applicable because we did not focus on EDs battery life. The column named Estimated Battery life for RD Using the Specifications in Each Paper presents the C battery life calculated from the kind of traffic, number of EDs, and technology datasheet specified in each compared paper. Although our method has better results, it should be taken into account that: [24] used 10 EDs, the samples per second are different for [20,22], and that the type of technology is different in practically all cases. The lesson learned is that by applying our method to RDs, their battery life will be extended in all the compared methods. 

We have shown that other methods used other WSN technologies. Next, we point several considerations about the applicability of our approach with other WSN technologies. We discarded IEEE 802.11ec because it was not frequently used in practical applications and nRF24 was a proprietary technology. We consider Bluetooth, Bluetooth Low Energy (BLE), and LoRa/LoRaWAN technologies. ZigBee and BLE are very low power consumption technologies. Bluetooth consumes more and LoRa/LoRaWAN was the most power-consuming technology, thus making LoRa/LoRaWAN a good candidate to extend the battery life of their devices. However, the LoRa/LoRaWAN supports star topology, which means we must focus on the battery life enlarging of the gateway (similar to ZigBee RDs). As we have shown in our practical experiments, our methodology will probably obtain good performance. More research must be done in order to assure that practical LoRa/LoRaWAN gateways could support sleep mode and the modification of its behavior to support our protocol and pseudocode of devices. With respect to Bluetooth, the master and the slaves in a piconet could be set in sleep mode. Our approach could be applied to the master. In both above technologies, the spanning tree calculation time will be zero because the topology of the WSN is a one-level tree. We do not know any practical standard Bluetooth scatternet. For this kind of WSN and mesh BLE WSN, our approach applies the implementation of the pseudocode of devices in the masters of the WSN. 

We used the XBee S2C device because it was the one in our lab (we have a limited number of devices). This did not make it possible to implement a particular realistic application. Moreover, for implementing our approach in a particular application, the firmware of the XBee S2C devices must be implemented (which can only be done by the manufacturer). The Digital Multi-meter JHS MY-64 was used for measuring current and voltage, though it is not a high precision tool. For that reason, we had to undertake a lot of measures to mitigate their variability and obtain average values. 

## 6. Conclusions

The ZigBee wireless sensor network contemplates the sleep mode of the operation for end devices but it does not contemplate a similar mode of operation for the Coordinator and the routers. This is because they have a higher responsibility than end devices. The Coordinator is in charge of configuring the wireless sensor network with other networks and the routers must find the routes to reach the Coordinator. We have proposed that the Coordinator and the routers include the sleep mode of operation when sensors deliver data periodically (sampling period) and the Administrator of the wireless sensor network could specify a global value for that period of time. We show that it can work for any topology of the wireless sensor network because, at the beginning of the sampling period, the routers and the Coordinator obtain information about the wireless sensor network spanning tree from the routing protocol. Moreover, we showed that practical experiments allow the enlarging of the battery life of the routers. The code to program or include in the firmware of the routers and the Coordinator is very simple, so it can be programed in a wide range of devices in the market. 

Our objective has been reached. Our proposal guarantees, in most of the analyzed cases and with the exposed conditions, an enlargement of router and Coordinator battery life for the ZigBee commercial wireless sensor network. In general, the value of the sampling period must be calculated taking into account the amount of end devices (the amount of messages to be forwarded by routers and the C), routers (a careful study of coverage among them must be realized in order not to augment the problems in the share wireless channel), and the structure of the resulting spanning tree (depending on the structure—depth and height—the routers could take more advantage of sleep mode). 

We are aware that more studies on battery life are needed, both from a theoretical and practical point of view. We are interested in the analysis of coordination among routers and the Coordinator; in particular, the analysis of new procedures that could coordinate the routers in order for them to enter sleep mode at different instants of the sampling period. Analysis of the practical influence of the wireless channel conditions is another interesting topic; in particular, we can take advantage of our previous work in the analysis of the wireless channel to estimate proactively the wireless conditions in order to take advantage of the buffering of messages in the routers (sending them when the wireless channel conditions are favorable). Finally, a more detailed study of the influence of ZigBee control traffic must be analyzed in order to optimize the overhead of control communication and the influence on how the transition to sleep mode could be done more quickly.

## Figures and Tables

**Table 1:** Pseudocode for our proposal.

**Pseudocode of *ED_i_***
***ReadConfigurationVariables*(*T*, *t_s_*)*;*** // *Administrator sets them* *Repeat* ***t*_1_** **= *ReadInternalClock* ();** // *Beginning of ton_i_* *D_i_* = *Data Sensing*; *Send* (*D_i_*); *Receive* (*Ack_i_*); ***t*_2_** **= *ReadInternalClock* ();** // *End of ton_i_* ***Sleep*** **(*t_s_* − (*t*_2_ − *t*_1_));** // *Beginning of s_i_* *Until* (*T--*) == 0; *//*
**Pseudocode of *RD_i_* (and the C)**
***//*** ***ReadConfigurationVariables*(*T*, *t_s_*);** // *Administrator sets them* *Repeat* ***t*_1_** **= *ReadInternalClock* ()*;*** // *Beginning of ton_i_* ***WaitSpanningTreeGenerated*** **();** // *Routing algorithm generates it* ***ComputeC*(*RD_i_*);** // (*or the C*) *to distribute vector M* *(Figure 3)* *For (p* = 1*, p* = *N(RD_i_), p*++) { // *Receive and send ACK* (*for each child of RDi*) *Receive* (*CD(RD_i_*)*_p_, D_p_*); *Send* (*CD*(*RD_i_*)*_p_, ACK_p_*); *Forward* (*Parent, D_p_*); *Receive* (*Parent, ACK_p_*); } ***t_2_*** **= *ReadInternalClock* ();** // *End of ton_i_* ***Sleep*** **(*t_s_* − (*t*_2_ − *t*_1_));** // *Beginning of s_i_* *Until* (*T--*) == 0; *//*

**Table 2:** Summary of *S_i_* obtained synthetic values.

Use Case	*o_i_* [*tu*]	*m_i_*	$t^{\prime }x_{i}\;[tu]$	Average Rounded *s_i_* [%]
*Ideal*	0 (fixed)	1	1	98
		2		96
		5		90
		10		80
		20		60
		30		40
*Acceptable*	Random in [0–1]	1	1	98
		2		92
		5		85
		10		70
		20		40
		30		15
*Acceptable* (*few EDs*)	Random in [0–2]	1	1	96
		2		90
		5		80
		10		60
		20		20
		30		0
*Acceptable* (*restrictions for large frame size*)	Random in [0–1]	10	1	70
		10	2	70
		10	3	30
		10	5	0
*Unacceptable* (*many EDs, EDs not synchronized or noisy network*)	Random in [0–3]	1	1	95
		2		90
		5		78
		10		50
		20		0
		30		0

**Table 3:** Specifications of current (Amp) in XBee S2C according to its states.

State	Amp
Operating current (Transmitting)	45 mA (+8 dBm, boost mode) 33 mA (+5 dBm, normal mode)
Operating current (Receiving)	31 mA (boost mode) 28 mA (normal mode)
Power-down current (sleep mode)	1 µA @ 25 °C

**Table 4:** Estimated battery life for different conditions and values of $\bar{s_{i}}$.

*m_i_*	$ton_{i}$ $[\%]|ton_{i}^{\prime }$	$\bar{s_{i}}$ $\;[\%]|{\bar{S}}_{i}^{\prime }$	Amperage Average	Estimated Battery Life
1	2 | 0.02	98 | 0.98	45 × 0.02 + 0.001 × 0.98 = 0.90098 mA	1100/0.90098 = 1220.89 h
2	8 | 0.08	92 | 0.92	45 × 0.08 + 0.001 × 0.92 = 3.6 mA	1100/3.6 = 305 h
5	15 | 0.15	85 | 0.85	45 × 0.15 + 0.001 × 0.85 = 6.75 mA	1100/6.75 = 162 h
10	30 | 0.30	70 | 0.70	45 × 0.30 + 0.001 × 0.70 = 13.50 mA	1100/13.50 = 81.48 h
20	60 | 0.60	40 | 0.40	45 × 0.60 + 0.001 × 0.40 = 27 mA	1100/27 = 40.74 h
30	85 | 0.85	15 | 0.15	45 × 0.85 + 0.001 × 0.15 = 38.25 mA	1100/38.25 = 28.75 h
No sleep mode	100 | 1	0 | 0	45 × 1 = 45 mA	1100/45 ~24.5 h

**Table 5:** Estimated battery life for different conditions and values of $\bar{s_{i}}$.

*m_i_*	$ton_{i}$ $[\%]|ton_{i}^{\prime }$	$\bar{s_{i}}$ $[\%]|{\bar{S}}_{i}^{\prime }$	Amperage Average	Estimated Battery Life
1	2 | 0.02	98 | 0.98	13.7 × 0.02 + 4.9 × 0.98 = 5.076 mA	1100/5.076 = 216.7 h = 9.02 days
2	8 | 0.08	92 | 0.92	13.7 × 0.08 + 4.9 × 0.92 = 5.604 mA	1100/5.60 = 196.2 h = 8 days
5	15 | 0.15	85 | 0.85	13.7 × 0.15 + 4.9 × 0.85 = 6.22 mA	1100/6.22 = 176.8 h = 7.3 days
10	30 | 0.30	70 | 0.70	13.7 × 0.30 + 4.9 × 0.70 = 7.54 mA	1100/7.54 = 145.88 h = 6 days
20	60 | 0.60	40 | 0.40	13.7 × 0.60 + 4.9 × 0.40 = 10.18 mA	1100/10.18 = 108.05 h = 4.5 days
30	85 | 0.85	15 | 0.15	13.7 × 0.85 + 4.9 × 0.15 = 12.38 mA	1100/12.38 = 88.85 h = 3.7 days
No sleep mode	100 | 1	0 | 0	13.7 × 1	1100/13.7 ~80.29 h = 3.3 days

**Table 6:** Qualitative comparison with other methods.

Method	Technology Testbed	Kind of Traffic	Battery Life Specified in Each Paper for Each ED	Estimated Battery Life for RD Using the Specifications in Each Compared Paper
[30]	NRF24 (2.4 GHz), PIC24FJ256, (10.8 v/3100 mAh battery)	Not specified	45 h 33 m	Not possible because kind of traffic was not specified in the compared paper
[27]	NRF24 2.4 GHz, PIC24FJ256, (10.8 v/3100 mAh battery)	Not specified	43 h	Not possible because kind of traffic was not specified in the compared paper
[24]	CC1101 (828 MHz), C8051, (3.6 v/1100 mAh battery)	3 samples per day	10.22 years	~4.9 years (~1 sample per 28,800 s)
[22]	XBEE S2, nRF24, AtMEGA 328P, (3.7/1000 mAh battery)	160 samples per minute	341 h	169 h (~300 samples per 100 s)
[20]	XB24, LPC824, 3v3 (USB, battery not specified)	1 sample per 10, 20 or 30 s	Not specified	130.9 h (10 samples per 100 s) (supposed 1100 mAh battery)
Ours	ZigBee XBEE S2C	1 sample per 10 s	Not applicable	216.7 h (1 sample per 100 s)
